# Italian Field Hospital Experience in Mozambique: Report of Ordinary Activities in an Extraordinary Context

**DOI:** 10.1017/S1049023X22000772

**Published:** 2022-08

**Authors:** Daniela Sacchetto, Mario Raviolo, Silvia Lovesio, Flavio Salio, Ives Hubloue, Luca Ragazzoni

**Affiliations:** 1. Disaster Medicine Service 118, ASL CN1, Levaldigi, Cuneo, Italy; 2. CRIMEDIM, Center for Research and Training in Disaster Medicine, Humanitarian Aid, and Global Health, Università del Piemonte Orientale, Novara, Italy; 3. Università degli Studi di Torino, Torino, Italy; 4. World Health Organization (WHO), Geneva, Switzerland; 5. Research Group on Emergency and Disaster Medicine (ReGEDiM) Vrije Universiteit Brussel, Brussels, Belgium

**Keywords:** cyclone, emergency medical team, field hospital, Mozambique

## Abstract

On March 15, 2019, Cyclone Idai made landfall near the port city of Beira in central Mozambique causing significant casualties and serious damage to infrastructure. The Emergency Medical Team Type 2 – Italy Regione Piemonte (EMT2-ITA) was deployed approximately two weeks after the disaster to support the country in need, providing essential medical and surgical care.

The EMT2-ITA staff was composed of 77 team members including two rotations and integrating local staff. A total of 1,121 patients (1,183 triage admissions) were treated during the 27 days of field hospital activity; among all the admissions, only few cases (17; 1%) were directly or indirectly attributed to the disaster event. Only three cases of cholera were confirmed and transferred to one of the treatment centers set up in Beira. The EMT2-ITA performed a total of 62 surgical operations (orthopedic, gynecological, general, and plastic surgery), of which more than one-half were elective procedures.

The objective of this manuscript is to report the mission of the EMT2-ITA in Mozambique, raising interesting points of discussion regarding the impact of timing on the mission outcomes, the operational and clinical activities in the field hospital, and the great importance to integrate local staff into the team.

## Specific Event Identifiers:

- **Event Type**: Cyclone
- **Event Onset Date**: March 15, 2019
- **Location of Event**: City of Beira, Mozambique
- **Geographic Coordinates**: lat = -19.84361; lon = 34.83889; elevation above sea level = 14m
- **Dates of Observation Reported**: March 23 – April 24, 2019
- **Response Type**: Medical Relief

## Introduction

In the early hours of March 15, 2019, Cyclone Idai made landfall near the port city of Beira, home to 500,000 people, capital of Sofala Province, in the central region of Mozambique. Approximately 90% of the city of Beira was destroyed.^1^ The cyclone, described by the United Nations (New York USA) as one of the deadliest storms on record in the Southern Hemisphere,^2^ caused a total death toll of more than 1,000 people (602 deaths in Mozambique, 344 people in Zimbabwe, and 59 people in Malawi)^3^ and left 2.6 million people in need of humanitarian assistance.^4^ Moreover, on March 27, the Ministry of Health (MoH; Maputo, Mozambique) declared a cholera outbreak, and through May, the number of cumulative cases increased to 6,766 with eight deaths.^5,6^

Under these circumstances, the Emergency Medical Team Type 2 – Italy Regione Piemonte (EMT2-ITA) was deployed approximately two weeks after the cyclone made landfall in Beira to support the country in need providing essential medical and surgical care. The EMT2-ITA is a classified World Health Organization (WHO; Geneva, Switzerland) Emergency Medical Team (EMT) Type 2, which is an “in-patient surgical emergency care” unit^6^ able to assure 24-hour operativity, to provide seven major/fifteen minor surgical operations per day, to manage 100 out-patients and 20 in-patients per day, and to remain totally self-sufficient from a logistical point of view.

The objective of this manuscript is to report the first mission of the EMT2-ITA after the WHO classification, focusing on the operational and clinical activities in the field.

## Sources

Data regarding the field hospital clinical activities were collected by the field hospital paper patient records and surgical procedures logbook filled by EMT2-ITA personnel during the entire mission. The purpose of this chart review was to depict a detailed picture of the clinical activity of the field hospital: the anonymous data of the patient records were manually entered into a data collection form (Microsoft Excel spreadsheet; Microsoft Corp.; Redmond, Washington USA) following specific coding rules to detect the variables of interest. The data collector was a medical doctor in order to recognize medical jargon and to reduce the risk of misinterpretation of chart entries or notes. The resulting electronic database was reviewed by two other professionals with experience in chart review studies. In case of missing data, these were clearly stated with a “not available” (N/A) value. The database was analyzed with Stata 15.1 (StataCorp LLC; College Station, Texas USA).

The study protocol was submitted to the Ethics Committee at Ospedale Maggiore della Carità in Novara, Italy and obtained its review approval (protocol number 258/CE).

## Observations

On March 20, 2019, the European Union Civil Protection Mechanism (EUCPM; European Commission; Brussels, Belgium) was activated following an official request for assistance of the Mozambique National Authorities (MNA; Maputo, Mozambique).^7^ The day after on March 21, the EUCPM sent a request to the Italian Civil Protection Department (Rome, Italy). According to the needs and the resources available within the European voluntary pool, the EMT2-ITA was dispatched to Mozambique.

**Figure 1.**
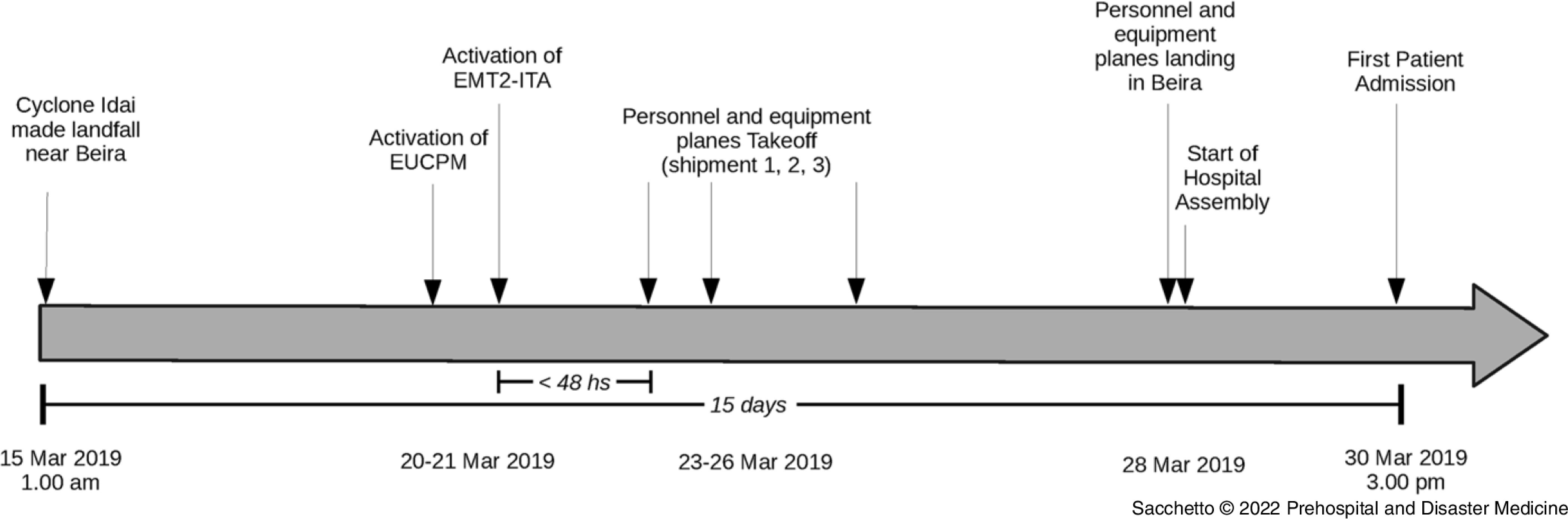
Timeline from Cyclone to Operational Deployment. Abbreviations: EUCPM, European Union Civil Protection Mechanism; EMT2-ITA, Emergency Medical Team Type 2 – Italy Regione Piemonte.

On March 30, 2019 at 3:00pm local time, the EMT2-ITA was the first EMT Type 2 to become operational among the 13 different international EMTs that arrived in the country. Figure 1 shows the timeline of the mobilization phase of the EMT2-ITA from Italy to Mozambique.

As requested by the MNA, the EMT2-ITA was set up within the courtyard of the Central Hospital of Beira (CHB; Beira, Mozambique), which is one of the three tertiary-care referral hospitals of the country, serving more than eight million people from Sofala Province and from the central region of Mozambique.^8^ Since the seven operating theatres (OTs) of the CHB were damaged and not functional from the day of the cyclone,^9^ the objectives of the EMT2-ITA were: (1) to support the CHB in its role as a referral hospital for surgical care within its area of reference; and (2) to become the receiving facility within the referral system established for the other international EMTs deployed in the region.

The EMT2-ITA consisted of:

1. A total of 77 team members, including a first group of 56 people and a subsequent rotation of 24 people; three people ensured the continuity of the operations by extending their stay for the whole deployment period (27 days). The second group was integrated by local staff, including medical doctors, surgeons, and nurses for the clinical activities and technicians for the logistics ones. In details, the EMT2-ITA was composed of 58 health care professionals (29 medical doctors, including two team leaders and one deputy team leader; 27 nurses; one x-ray technician; and one midwife) and 19 operation support personnel (one engineer; four electricians; two water, sanitation, and hygiene [WASH] experts; and 12 logisticians). Table 1 shows the structure of the staff working each shift in the different departments of the field hospital.
  **Table 1.** Structure of Staff Working Each Shift in the Field Hospital.

Field Hospital Department	Personnel Type per Shift (n)
Triage and Registration	Nurse (1)
Emergency Room	Nurse (1)
	Emergency Physician (1)
	Pediatrician (1)^a^
	Gynecologist (1)^a^
	Midwife (1)^a^
Shock and Critical Care Room	Nurse (1)
	Anesthesiologist (1)
Post-Anesthesia Care Unit and Sterilization Room	Nurse (1)
Operating Theatre	Nurse (2)
	Surgeon (2)
	Anesthesiologist (1)
Isolation Room	Infectious Disease Medical Doctor (1)^a^
X-Ray and Laboratory	Nurse (1)
	X-Ray Technician (1)^a^
In-Patient Wards	Nurse (1)

Note: Shifts were typically two shifts per day, eight hours.

a On-call availability for some specialists (single figures in the team).
2. A temporary structure of nine tents with a total of 26 beds. As depicted in Figure 2, the functions of the nine tents were: (1) triage and registration; (2) emergency room (ER); (3) shock and critical care room (four beds) with a gynecological corner; (4) post-anesthesia care unit and sterilization room; (5) OT; (6) isolation room for suspected or confirmed infectious disease (two beds); (7) x-ray and laboratory; and (8)(9) two in-patient wards (10 beds for each tent).
  **Figure 2.**
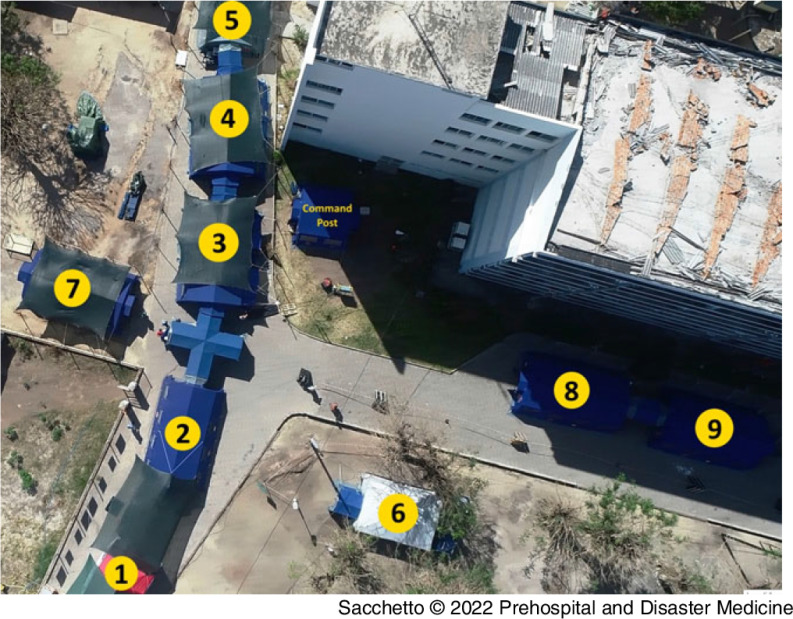
Deployed Field Hospital within the Courtyard of the Central Hospital of Beira: (1) Triage and Registration; (2) Emergency Room (ER); (3) Shock and Critical Care Room with Gynecological Corner; (4) Post-Anesthesia Care Unit and Sterilization Room; (5) Operating Theatre (OT); (6) Isolation Room for Suspected or Confirmed Infectious Disease; (7) X-Ray and Laboratory; and (8)(9) Two In-Patient Wards.

During the 27 days of EMT2-ITA activity in Beira, a total of 1,121 patients were treated. Given the several re-presentations, a total of 1,183 triage admissions were recorded.

The ages of the patients were highly variable (mean = 36.64, standard deviation = 18.80), ranging from newborn to 93 years. The distribution was 53% (596) of patients were females, within the adult age group (18-64). Among all the admissions, only few cases (17; 1%) were directly or indirectly attributed to the disaster event and they presented to the facility during the first 10 days of activity. Three cases of cholera were confirmed and transferred to one of the three treatment centers set up in Beira by Doctors Without Borders/Médecins Sans Frontières (MSF; Geneva, Switzerland). Almost all the admissions (1,071; 91%) ended with discharge the same day of admission and more than one-half of them (705; 60%) were due to chronic conditions. Table 2 summarizes the demographic and clinical characteristics of the patients presented to the EMT2-ITA in Mozambique.

**Table 2.** Summary of Demographic and Clinical Characteristics of Patients Presented to the EMT2-ITA

	Total n (%)
**Age**	
0 y	12 (1.0%)
1 - 4 y	73 (6.2%)
5 - 17 y	87 (7.3%)
18 - 64 y	931 (78.7%)
> 65 y	79 (6.7%)
Unknown	1 (0.1%)
**Gender**	
Female	752 (63.6%)
Male	431 (36.4%)
**Triage Category** ^a^	
Green	1071 (90.5%)
Yellow	49 (4.1%)
Red	7 (0.6%)
Blue	1 (0.1%)
N/A	55 (4.7%)
**Diagnostic Category**	
Gastrointestinal	178 (15.0%)
Musculoskeletal	174 (14.7%)
Obstetrics/Gynecology	160 (13.5%)
Miscellaneous	103 (8.7%)
Infectious	86 (7.3%)
Trauma	85 (7.2%)
Respiratory	84 (7.1%)
Surgical	75 (6.3%)
Genitourinary	62 (5.2%)
Neurological	62 (5.2%)
Cardiovascular	50 (4.2%)
Ear/Nose/Throat	40 (3.4%)
Dermatology	17 (1.4%)
Ophthalmology	5 (0.4%)
N/A	2 (0.2%)
**Outcome**	
Discharged	1071 (90.5%)
Hospitalized	46 (3.9%)
Transferred	47 (4.0%)
Death	3 (0.2%)
Left	1 (0.1%)
N/A	15 (1.3%)
**Cyclone Relation**	
Not Related	1106 (93.5%)
Related	10 (0.8%)
Indirectly Related	7 (0.6%)
N/A	60 (5.1%)

Abbreviation: EMT2-ITA, Emergency Medical Team Type 2 – Italy Regione Piemonte.

a Color codes according to this encoding: Green = Standard, Yellow = Urgent, Red = Immediate, and Blue = Expectant.

As shown in Table 3, the EMT2-ITA performed a total of 62 surgical operations on 59 patients (three patients were operated on twice). More than one-half were an elective procedure. Most of the surgeries were orthopedic (27; 44%), followed by gynecological (19; 31%), general (10; 16%), and plastic surgery (5; 8%).

**Table 3.** Summary of the 62 Surgical Operations Performed by the EMT2-ITA

	Total n (%)
**Surgical Session**	
Morning	26 (41.9%)
Afternoon	23 (37.1%)
Morning/Afternoon	3 (4.8%)
Afternoon/Night	3 (4.8%)
Night	3 (4.8%)
N/A	4 (6.5%)
**Team**	
Italian	33 (53.2%)
Mixed	16 (25.8%)
Local	12 (19.4%)
N/A	1 (1.6%)
**Type of Operation**	
Elective	33 (53.2%)
Emergency	25 (40.3%)
N/A	4 (6.5%)
**Specialty**	
Orthopedics	27 (43.6%)
Gynecology	19 (30.6%)
General Surgery	10 (16.1%)
Plastic Surgery	5 (8.1%)
Orthopedics + General Surgery	1 (1.6%)
**Anesthesia**	
Spinal	22 (35.5%)
General	9 (14.5%)
Spinal + Sedation	6 (9.7%)
Peripheral Block + Sedation	5 (8.1%)
Peripheral Block	4 (6.5%)
Sedation	4 (6.5%)
Spinal + Peripheral Block	2 (3.2%)
N/A	10 (16.1%)
**Outcome**	
Hospitalized	43 (69.4%)
Discharged	9 (14.5%)
N/A	10 (16.1%)

Abbreviation: EMT2-ITA, Emergency Medical Team Type 2 – Italy Regione Piemonte.

The mission ended on April 25, 2019 with the last three patients admitted during the night; all the field hospital equipment was handed over to the CHB with a formal donation to the MNA.

## Analysis

Describing the first mission of the EMT2-ITA after the WHO classification, this report raises interesting points of discussion regarding the impact of timing on the mission outcomes, the field hospital operativity, and the great importance to integrate local staff into the team.

First, the impact of mobilization timing on the clinical presentation: the EMT2-ITA became operational 15 days after the event due to the delayed request for assistance and the long travel time. Only few patients with specific disaster-related injuries had access to the EMT2-ITA, in contrast to many patients coming to the field hospital for routine medical care. Similarly, the surgical activity was predominantly elective. As largely described in the literature, this clinical presentation is very typical in the aftermath of disasters. In 2005, Bar-Dayan and colleagues^10^ reported only minimal effort in treatment of earthquake-related injured victims (90% of patients with non-traumatic illnesses) when describing the Israeli Defense Forces mission in Duzce, Turkey after the earthquake of 1999. Bar-On, Peleg, and Kreiss, in their recent book *Field Hospital: A Comprehensive Guide to Preparation and Operation*,^11^ analyzed the evolution of health needs during a generic disaster, reporting the decrease of surgical cases in spite of medical cases in the first seven and fourteen days after the event. In addition, the introduction of chronic disease care and rehabilitation in the WHO checklist of the minimum standards for EMTs Type 2^12^ is further proof that EMTs must be ready to cope with daily emergencies and routine activities during their missions.

Additionally, the EMT2-ITA was embedded into the CHB, whose entire surgical floor was flooded, the roof damaged, the equipment destroyed, and the power supplies cut off after the cyclone. This meant that all the surgery rooms, except one for C-sections, were not functional. Therefore, apart from the timeline, it is clear the essential role played by the EMT2-ITA was replacing the surgery capacity and facing the routine patient load of the local hospital.

Even though the EMT2-ITA became operational just three days after the declaration of the cholera outbreak, only three cases presented to the field hospital and all of them arrived during the first week of activity. The epidemic curve of cholera cases reported by the WHO^13^ shows a significant rise of cases in the first weeks after the cyclone, reaching the peak on April 8, 2019, with a gradual decrease in the following days. From April 3 to April 9, the MoH conducted a mass-vaccination campaign that reached more than 800,000 people (90% of the target population).^5,6,13^ Moreover, the MNA and international partners immediately set up several treatment centers and established a WASH taskforce, which then quickly implemented interventions to ensure the provision of safe WASH facilities for the local population.^14^

Secondly, the under-utilization of the in-patient wards: the small number of patients needing hospitalization after treatment were admitted directly inside the CHB (hospitalization rate = 4%). Admitting patients to the local hospital shows again the important complementary role of the EMT2-ITA for the CHB. According to this situation, it is evident the importance of the information provided during the request of assistance that, since it was done one week after the cyclone, should have reported data about the local hospital and the role expected for an EMT Type 2.

An equally important aspect regarding the field hospital management is that, although the EMT2-ITA is ready to assure field hospital operativity 24/7, as requested by minimum standards established by WHO classification, during the Mozambique mission, no patients presented at night as transportation became much more difficult after dark. To adapt to this night in-activity, the personnel shifts were scheduled only during the day, while at night (from 12:00pm to 8:00am), on-call availability was organized for the surgery room and a medical doctor supervised the ER and the triage tents in case of patients coming.

Thirdly, the integration of the local staff in the team composition during the rotation of the personnel of the EMT2-ITA: this integration was really remarkable because, on one side, it allowed to limit the number of professionals coming from Italy and, on the other side, it ensured an effective training about the correct use and maintenance of the entire field hospital equipment before the final donation of it to the MNA.

## Conclusion

The data collected show once again that the main role of an EMT becoming operative several days after a disaster event is the full commitment for elective activities to support and maintain the ordinary health care capacity of the affected country; this is confirmed by the final report of WHO^15^ regarding the Mozambique mission, reporting that the 82% of patients treated by EMTs were not related to the Cyclone Idai.

This is an important conclusion, driven by this and several other^6^ direct experiences in the field, that must be kept in mind for different levels of the disaster response and management.
